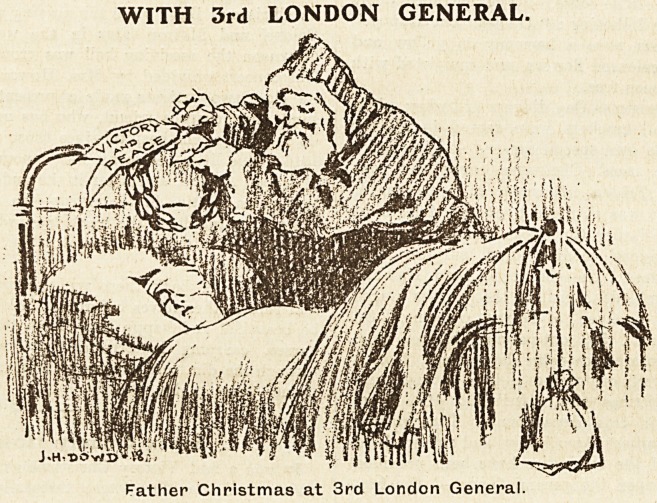# Our Hospitals' Christmas, 1918

**Published:** 1919-01-11

**Authors:** 


### OUR HOSPITALS' CHRISTMAS, 1918.

### "Dreadnought" Hospital, Greenwich.

TELEGRAM FROM H.M. THE KING.

The Sailors, both Naval ratings and the equally gallant men of the Merchant Service, thoroughly enjoyed their Christmas in the "Dreadnought" Hospital. The wards were as usual decorated in true Christmas fashion, festoons of holly and ivy being seen in every ward and corridor of the old building. On Christmas morning every patient found a gift by his bedside, and after breakfast and service in the chapel, dinner was served in the wards. Turkey, ham, beef, and plum-pudding were the principal items of the feast, to which were added mince-pies, fruit, sweets, etc. Those not too ill to be up and about sat round the festive board, while those who were confined to bed and yet could eat Christmas fare had it brought to them. There were others, however, who could not join in these good things, who were tended and cared for by the nurses generally. The old English customs were enhanced by the presence of the Mayor of Greenwich and Deptford, Mr. Alderman J. Stone, J.P., and Col. W. A. Wayland, J.P., whose unremitting interest in the hospital for many years is well known They wore their robes of office as a compliment to the men to whose courage and disregard of danger so much is due, and in every ward they said kindly words of praise and cheer to those assembled. The following telegram was received from H.M. the King, and was read by the Mayor to the patients :

> Another Christmas has come round and we are no longer fighting. God has blessed your efforts, the Queen and I offer you our heartfelt good wishes for a Happy Christmas and many brighter years to come. To the disabled sick and wounded we send a special greeting, praying that with returning health you may be comforted and cheered by the vision of those good days of peace for which you have sacrificed so much.
>
> George R.I.

During the afternoon concerts were held in some of the wards. Plenty of tobacco and plenty of pipes, also fruit and other good things beguiled the remainder of the Christmas Day, which was surely one of the most cheery and inspiring that has been passed in the old "Dreadnought."

### Christmas Day at St. Mary's.

On Christmas morning every patient received a present. The children had huge stockings filled with good things. All men well enough were allowed to smoke. Carols were sung in the Chapel at the morning service by the hospital choir, and in the hall, where the accompaniments were played by Miss Florence Williams, acting sister of the Cambridge Ward. The patients had turkey and plum-pudding for dinner. The ward decorations were both ingenious and artistic. Two prizes were offered for the best decorated wards. The first was won by Grafton, a Japanese garden—a veritable garden with a pond, a river, a fancy bridge with a Japanese lady fishing from it—all kinds of festoons and Japanese umbrellas. Cambridge, as "The Blue Bird" won the second prize. De Hirsch, the children's ward, looked so pretty in pink and white; all the decorations were purely and simply for the little people. The Christmas tree caused great delight. The ward teas were at 4 p.m., and each patient was allowed to have one visitor. The tables were beautifully decorated and well supplied with good things. The sisters worked hard to make the evening a great success. There were four troupes of residents, men and women, pierrots, the "'Orrible 'Owlers," Toy Symphony Troupe, "The Rest Cure," a playlet in one act; "Vera's Revenge," Cinema by St. 'Mary's Famous Film Firm. They were one and all "second to none." The patients enjoyed the novelty of seeing their own doctors in fancy costumes. There was no lack of entertainment—all the wards had plenty of willing helpers. The nurses played or sang, and eight o'clock came only too soon. Next the sisters' dinner took place on Boxing Day and was followed by the nurses' on the 28th. Both rooms were decorated, and all thoroughly enjoyed their annual gathering.

### WITH 3rd LONDON GENERAL.

Father Christmas at 3rd London General.

### King's College Hospital.

This year the welcome changed circumstances made it possible to prepare for the festive season on a more liberal scale than during the past four years. In the Twining Ward there were scarlet lamp shades with scarlet Japanese lanterns, the lights over the beds were surrounded with clusters of ivy and festoons of berberis, and large vases of berberis with red chillies and variegated holly further adorned the ward. In the Lister Ward the centre lights were made to represent old-fashioned lanterns in red and black, and the lights over the beds were of floral design. The general decoration of the ward consisted of flags, evergreens, and festoons of ivy and japonica, and on the centre ward table was to be seen a winter scene of a snow-clad cottage. The Lonsdale Ward was decorated with holly, the bed lights being shaded with red crinkled

The first article appeared in the "Hospital" of January 4.

**OUR HOSPITALS' CHRISTMAS, 1918—(*continued*).**

paper. The frames of certain fractured femur beds were decorated with flags and the mantlepieces bedecked with fairy lights. The prevailing colours in the Trundle and Waddington Wards were flame and gold. The centre fireplaces, tables, &c., showed gold flowers, smilax and fairy lights, and in the corridor linking up the two wards was a daintily arranged illuminated cosy corner. Matthew Whiting Ward was decorated in yellow and gold, the lights representing water lilies. There were choice yellow and bronze chrysanthemums, and in the centre of the ward was a very fine bush of holly. The Storks Memorial Ward was unfortunately in quarantine owing to a slight infectious outbreak. However, in spite of this unfortunate occurrence, the ward was prettily decorated. Over the head of each bed Father Christmas appeared, and the ward from one end to the other was one mass of suspended air balloons and birds of various colours. There was a Christmas tree 18 feet high laden with suitable gifts, and illuminated with electric lights. The gifts were distributed by Father Christmas, to the great delight of the little ones. The entrance doors to this ward bore the following : "Unclean but Happy." The observation wards were a harmony in yellow and orange, with golden coloured flowers, and completed with an illuminated Christmas tree.

At 12 o'clock on Christmas Day dinners of turkey, plum pudding, dessert, and crackers were served in all the wards, each having its own special carver, clad in special fancy costume. The usual Christmas party and concerts took place on Friday afternoon in every ward, where the ward sister was at home to receive friends of the hospital. Again Father Christmas was present, and members of the resident medical and surgical staff appeared in fancy dress. The chairman, Lord Hambleden, with Lady Hambleden, visited the various wards that afternoon, and received a hearty welcome. On Monday, the 30th inst., 360 out-patient children each received in the decorated casualty department, from a "Bran Tub," a parcel containing a bun, orange, and cracker. Father Christmas also distributed gifts from the Christmas tree in this department. Through the generosity of the Ladies' Association and other kind friends, the whole of the festivities have been provided without encroaching upon the general funds of the hospital.

### The Third London General Hospital.

PERHAPS the feelings with which we prepared for and kept Christmas this year at the Third London General Hospital were best summed up by the Colonel's Christmas card, containing his message of thanks to the staff for their co-operation during the past years, and illustrated by Lance-Corporal Dowd's exquisitely tender drawing of Father Christmas placing a laurel wreath, inscribed "Victory and Peace," above a sleeping patient.

It was the Victory Christmas, won for us by the shattered men whose light-hearted merriment and wonderful endurance were as astonishing to us on this fifth Christmas as they had been on the first : it was also almost certainly our last Christmas in the little world which the war had called into being, and which we had learned to love. So for many of us happiness was tinged with a vague regret, though outwardly there were no signs of sadness—far otherwise.

As the visitor entered the main door he was welcomed in by "A Merry Christmas"—scarlet letters upon a white ground, framed in laurel leaves—and the same welcome was repeated in the wards. Never had they been gayer, or borne more evidence to the ingenuity of patients and staff. Holy and evergreens, flags of the Allies, Chinese lanterns and umbrellas, festoons of brightly coloured paper, gaily tinted balloons and fairy lights, had all been requisitioned to aid in various schemes of decoration, while conspicuous in each ward was the beautiful floral basket sent by the Colonel's wife and daughters. And everywhere cunningly concealed sprigs of mistletoe awaited the unwary.

The days immediately preceding had been busy ones for the Ladies Committee, headed by Mrs. Bruce Porter and Lady Pearce Gould, and there were presents for everyone : "The Doings of Donovan," by that most brilliant and unassuming member of our staff, J. H. Dowd; leather wallets, sweets and cigarettes. Patients and staff alike received parcel or stocking, and to most of us the contents were not merely kind Christmas gifts, but mementoes to carry away into the Peace years of happy work and happier companionship.

Of Christmas fare there was no stint, patients and R.A.M.C. having their turkey and plum-pudding actually on Christmas Day, while the nursing staff dined with the Colonel and Matron later in the week. On Christmas afternoon the receiving hall was crowded for the excellent concert provided by Mrs. Howard Williams, and at its close two patients made a presentation to that most kind and generous friend, who has never seemed able to do enough for the hospital since those first days of August 1914. The little ceremony was honoured by the presence of Queen Amélie, and after it the audience trooped away to the tea parties in the wards—tea parties which were repeated throughout the week, and which were enlivened in many cases with music and songs, and even informal whist drives.

As we look back there will be many pictures which will reform themselves before our mind's eye, but all will be beautiful and happy ones, from the procession of the nurses, carrying lanterns and singing carrols, winding through the dim corridors on Christmas Eve to the V.A.D. dinner three nights later.

### Essex County Hospital.

IT was a real Victory Christmas at this hospital. The wards and corridors were tastefully decorated. Each ward had its Christmas tree, which in every case was laden with gifts. Carols were sung on Christmas Eve, and then patients were settled to sleep, to find on waking that Father Christmas, after a long absence, had left each one a parcel. The smallest child was not forgotten.

Great excitement prevailed during the morning watching the good things arrive for dinner, and when at 12 o'clock various members of the visiting and honorary staff carried in the turkeys, there was loud cheering. Then followed plum-pudding and other good things. There was plenty of everything for everyone. In the soldiers' wards there was a guessing competition with prizes as to the correct weight of their turkey.

During the afternoon there were hat-trimming competitions in each ward, which the Mayor and Mayoress, on there way round the hospital, judged, and gave prizes for the best one in every ward. It was a great treat when tea-time came to see once more large fruit cakes on each table, amongst other good things. From 4 p.m. until 7 p.m. Father Christmas, impersonated by one of the honorary staff, visited the wards to unload the trees. The small children who had never seen Father Christmas

**OUR HOSPITALS' CHRISTMAS, 1918—(*continued*).**

before were at first rather frightened, but his cheery smile soon made them happy. He forgot no one, and looked very tired when his task was finished. There were various concerts going on from time to time in the wards; and in some musical chairs, twirl the trracher, and other good old Christmas games provided much amusement until 10 p.m., when everyone looked tired. Before retiring, many hearty cheers were given for the Matron (Miss M. E. Jones), who has earned the love of both patients and staff. There was also great cheering for the cook, who had worked hard to make the event a success.

Concerts were held in the wards during the succeeding days by the day and night staff, and were greatly appreciated by patients and visitors. On Boxing night the maids had their Christmas dinner. Music and games followed, and each one finished up thoroughly happy.

The nurses' dinner was held on New Year's Eve. Each represented a book. A prize was given by the matron for the one who guessed most. The New Year was welcomed in by "Auld Lang Syne" as the clock struck 12. The sisters' dinner was on New Year's Night. Each had to be a proverb. Matron again gave a prize for the best. Everyone had a happy time and will remember their Victory Christmas.

### Northampton General Hospital.

THE seasonable festivities extended over seven days. A feature was the distribution of pocket-wallets made of leather as representative of one of the town's staple industries. Altogether 1,700 wallets were distributed to wounded soldiers in the district, and each was accompanied by a letter written by the Mayor of Northampton on behalf of the Northampton Allied War Fund. In the hospital on Christmas Day there were concerts and other amusements in the wards. On Boxing Day the Mayoress, who was accompanied by the Mayor, distributed presents from the Christmas tree, and a concert was given by the nursing staff. On Friday the entertainment of the patients was in the hands of Mr. W. Arnold, jun., and Mr. Matthew Arnold; on Saturday Councillor and Mrs. P. D. Lewis made themselves responsible for the festivities; on Monday, Mr. and Mrs. P. D. Wren; on Tuesday, Mr. and Mrs. L. Steinberg; and on New Year's Day, the members of the Olney Working Men's Club. On each of these days tea was provided, and there were concerts and competitions.

### Norfolk and Norwich Hospital.

ALTHOUGH the number of military patients was considerably less than at this time last year the festivities provided lacked nothing in interest compared with those of previous years. The decorations in the soldiers' wards consisted mainly of flags of the Allies, whilst in the civilian wards the sisters had arranged schemes of decoration which, if not so ambitious in colour and extent, were quite as effective from an artistic point of view. No public appeal was made, and few gifts of money and in kind were received. The arrangements for Christmas Day were very similar to previous years, Holy Communion and the usual morning services in the chapel being followed by an excellent Christmas dinner for all, the fare including turkey and plum pudding. The carving of the turkeys was undertaken by members of the honorary medical staff and other officials, whose presence added greatly to the enjoyment of the meal. During the afternoon the distribution of useful gifts from Christmas trees in three of the wards was followed by a special Christmas tea. Many of the wards arranged for their own scheme of entertainment during the afternoon, with the exception of one of the military wards, in which a most popular performance was given by the company from the local music hall. The heavy work associated with the reception of military patients has prevented the members of the nursing staff from giving entertainments on a large scale during the war, but this year the sisters and nurses presented an attractive programme in the Recreation Room on Boxing Day and the two following days. The mounting and dressing of most of the items, which were largely of a spectacular character, formed a noteworthy feature; songs and dances were followed by a tableau representing Britannia and her Allies, which brought to a close a performance which had provided keen enjoyment for over two hours. Great credit is due to the members of the nursing staff for the time and trouble spent in the preparation of this performance, in addition to their ordinary duties.

### North Staffordshire Infirmary.

THE day of the Christmas-box not being a thing of the past, the Secretary and House Governor (Mr. W. Stevenson) of the North Staffordshire Infirmary, Stoke-on-Trent, conceived the idea that an appeal for £1,000 for the infirmary might not pass unheeded. Accordingly he planned his campaign (after arranging for special fare for patients and staff, and for their entertainment on good old-fashioned lines) and by liberal use of the telephone he succeeded in half-finishing his task on Boxing Day. This was duly chronicled in the local press and further appeals made, with the happy result that he was enabled to write *fait accompli* to the project within the octave of the great festival. To garner £1,000 for the Infirmary funds, and to arrange, in conjunction with the matron and other officials, for the well-being of patients and staff so that ante-war conditions might once more prevail was the aim of the House Governer. He must regard the result as entirely satisfactory.

Unusually pretty and effective decorations of the wards by the sisters and nurses were carried out in friendly rivalry. Santa Claus unburdened his heavy load. Carols in the very early hours of "the day"; a well-attended service in the Chapel; the much-enjoyed turkey dinner; the etceteras following; the visits by members of the committee, honorary medical staff, honorary chaplains, with their families and patients' friends; the fancy-dress dance by the nurses; the children's ward Christmas-tree, and the stripping thereof by a splendid "Clown"; and the Punch and Judy show, all in turn tended to make this Christmastide memorable in the annals of the infirmary.

### Royal Infirmary, Preston.

FESTIVITIES commenced at the Queen Victoria and County of Lancaster Royal Infirmary on Christmas Eve, when the choir of St. Paul's Church sang carols in the corridors. The wards were tastefully decorated, and, as usual, in the children's ward there were two large Christmas trees. At 5 o'clock on Christmas morning the nurses sang carols. The patients, upon waking, found that Father Christmas had paid a visit, leaving suitable garments for all. The children's stockings, too, were filled with toys and useful articles. After the Christmas dinner, a conjuring entertainment was given by a friend of the institution, and was much appreciated. On Boxing Day the patients had their tea-party. On Friday evening the nurses had their Christmas dinner, after-

**OUR HOSPITALS' CHRISTMAS, 1918**—(*continued*).

wards proceeding to the theatre to witness the pantomime. The wards were relieved by the sisters, who had their Christmas dinner on Saturday evening; they also finished up the evening at the theatre. Festivities were continued on Monday evening, when a concert party gave an enjoyable vocal and humorous entertainment.

On Tuesday afternoon, Father Christmas, attired in a gorgeous robe, limped into the children's wards, where he was eagerly awaited. After greeting the little ones, he proceeded to strip the two large Christmas trees, and distribute the dolls and toys to the delight of all. Father Christmas will be long remembered by the children. In the evening the night nursing staff gave an entertainment, consisting of songs, humorous duets, and two funny sketches, entitled "Henpecked" and "Lodgings for Single Ladies." On Wednesday evening the pantomime "Beauty and the Beast," was presented by the day nursing staff. Several popular songs were introduced. The festivities ended on Friday night with a fancy-dress dance for the nursing staff. Some of the costumes were very original.

### Royal Albert Edward Infirmary, Wigan.

Christmastide at the Royal Albert Edward Infirmary and Dispensary, Wigan, was a season of real joyousness, thanks to the splendid efforts of the staff and the generosity of friends. On Christmas Eve members of the nursing staff cheered the hearts of patients by singing carols. The Chairman of the Board of Management (Mr. William Johnson, Junior), the Senior Honorary Surgeon (Dr. E. Hodkinson Monks), and the Matron (Miss M. K. Coggins), toured the wards on Christmas morning, and each patient received a useful present. To those who could partake of such fare, turkey, plum pudding, mince pies, sweets, fruit, etc., were given at dinner. The sixty-seven sick and wounded soldiers had a Christmas tree which contained many acceptable gifts. Enjoyable entertainments were provided by friends of the hospital, and the nursing and domestic staffs had fancy dress dances.

On Friday, the ceremony of dismantling the Chrismas tree in the children's ward, and the viewing of the wards and departments by visitors took place. The distribution of gifts to the children and various members of the staff was made by Lady Margaret Lindsay, who was accompanied by Lady Elizabeth Lindsay, daughters of the Earl of Crawford and Balcarres, P.C.

Mr. William Johnson (Chairman), in welcoming the lady representatives of the House of Haigh, paid tributes to their kindness.

Lady Margaret Lindsay, in dismantling the tree, said she was very glad to be present at such a gathering—the first of its kind for a long while. Lady Margaret expressed gratitude to the brave soldiers, sailors and airmen. The rôle of Father Christmas was well filled by Dr. A. Harper, a house surgeon. Tea was served to the visitors in the nurses home. Many were the testimonies to the excellence of the appearance and work of the hospital. A great feature was the beautiful scheme of decoration which reflected much credit upon the sisters and nurses. Flags, streamers, coloured lanterns, and lights were intermingled with holly, evergreens, imitation branches, birds, snow, etc., and produced a delightful effect. The organising of the festivities, etc, was largely the work of the matron (Miss M. K. Coggins), and to her and the members of the nursing staff great praise is due for the splendid results.

To the appeals made by the Chairman (Mr. Johnson), and the General Superintendent and Secretary (Mr. Hedley Lucas) on behalf of the Christmas fund, there was a successful response, a sum of £81 10s. (an increase of £38 upon that of last year) being obtained, in addition to many gifts in kind.

The conclusion of a round of happy events was the nurses whist drive and dance, useful prizes being kindly furnished by Mr. Johnson. A high-class concert given by local talented ladies and gentlemen, followed.

### 4th Southern General Hospital, Plymouth.

The Salisbury Road Hospital has, since the first year of the war, been justly proud of its Christmas festivities and fun, and the patients and staff agreed that the celebration of 1918 was the happiest and brightest of all. Everyone entered into the work right willingly, and the results were wonderful. To each patient was given a present, a shirt, a pair of socks, and a handkerchief; with each went a Christmas card, very cleverly designed by one of themselves, bearing a greeting from Father Christmas. After a short service in the Church Hut the men returned to find the table groaning under its load of Christmas fare. The sisters and staff served a dainty but bountiful tea for the patients and visitors; this was greatly appreciated by the men, as the personal gift of the nursing staff. Then came the Christmas tree, and more fun and frolic till bedtime—a very late bedtime indeed.

### Royal Aberdeen Hospital for Children.

For the second time the patients of the Royal South Aberdeen Hospital for Sick Children largely owed their big Christmas tree and tea parties to the work and generosity of the wounded soldiers. At the Durris House Auxiliary Hospital, where Mrs. Baird, who takes great interest in the children, is commandant, the men have worked hard. Shortly before Christmas they held a sale, with such success that last year £24 was handed over to the matron, and this year £30.

The custom at the Children's Hospital has been for each little patient to be allowed a choice of one gift (and if funds permit every effort is made to gratify this particular wish), and a second gift comes as a surprise. But with toys at such a high price it was feared that the tree might not be as well laden as usual. Once again, however, the wounded men stepped into the breach, and could they have seen the circle of eager, expectant little faces drawn up round the tree ready for stripping on Christmas Day they would have been well rewarded for their labours.

### Chelsea Hospital for Women.

The annual entertainment and tea arranged by the Ladies' Committee took place on Saturday, the 4th inst. The entertainment consisted of an interesting performance by Mr. Thomas Burrows as conjuror and ventriloquist, and was much appreciated by a large audience. The wards are again devoted to the treatment of the diseases to which women in particular are liable. Over 400 wounded officers have been received during the past fifteen months. When funds are available for building a new nurses' home and for maintaining the patients who can then be admitted, the full value of the hospital to suffering women will be realised. The Convalescent Home at St. Leonards-on-Sea is a great factor in their full restoration to health.

(Concluded on page 322.)

### Our Hospitals' Christmas, 1918 (*concluded from p.* 320).

### Alton Crippled Children's Hospital.

THE staff threw themselves wholeheartedly into the enjoyable work of preparation for Christmas festivities. The wards were decorated with taste, enhanced, from the children's point of view, by fine Christmas trees bearing glorious burdens. "The Day" was a day of hard but completely enjoyable work for the staff, and of almost delirious pleasure for the patients. Dr. Gauvain, medical superintendent, officiated as "Father Christmas" with immense success, and was very ably seconded by "Mrs. Christmas" (Dr. Wood). The gifts included chocolates from Queen Alexandra and new shilling-pieces from a donor. During the afternoon two parties of nurses, in costume, gave delightful entertainments.

### Nurses in "Revue" at Winsley Sanatorium.

CHRISTMAS DAY at Winsley Sanatorium will long be remembered by the patients, as from early morning till late at night one continual round of surprises and pleasures was enjoyed by all. The preparation had been in hand for quite a fortnight by the staff, and on Christmas Eve patients decorated the dining-hall and block corridors with flags, bunting, evergreens, etc.

Dinner was done full justice to by all. There was no shortage of Christmas fare. After dinner, good humour prevailed on every hand, and three hearty cheers, led by the youngest male patient, were given to the doctor, matron, sister, and nurses, not forgetting the cook. Great sport was provided for the children with the ever-fresh bran-tub, into which each child had a dip and fished up a present.

At four o'clock all had tea, which consisted of many good things, and was enlivened by music by Sister Brown and Nurse Brooks. Then a successful whist-drive took place and prizes were given, followed by an excellent concert, to which both patients and staff contributed. Two humorous songs sung by Dr. Melville Rees were enjoyed by everyone. But the chief attraction of the evening was a "Revue" entitled "Allies at Winsley," which formed the second part of the programme. This was performed by Sister Patricia Brown and the nurses. The dresses were beautiful, and the rollicking choruses were heartily sung by all. Perhaps the most realistic item was an Indian dance by Sister Brown and her two small attendants—the Indian costumes provided by matron—were typical. Nurse E. Brooks delighted everyone with her Irish songs and jigging.

## Figures and Tables

**Figure f1:**